# The Influence of Demographic Characteristics, Pre-Existing Conditions and Laboratory Parameters on Postoperative Hemorrhage After Brain Tumor Surgery

**DOI:** 10.3390/life15121941

**Published:** 2025-12-18

**Authors:** Anatoli Pinchuk, Nikolay Tonchev, Anna Schaufler, Claudia A. Dumitru, Klaus-Peter Stein, Belal Neyazi, I. Erol Sandalcioglu, Ali Rashidi

**Affiliations:** Department of Neurosurgery, Otto-von-Guericke-University Magdeburg, 39120 Magdeburg, Germany

**Keywords:** age, comorbidities, demographic factors, postoperative hemorrhage, brain tumors

## Abstract

**Background:** Postoperative hemorrhage (POH) is a rare yet serious complication of cranial surgery, potentially resulting in extended hospitalization, neurological impairment, or death. Existing predictive models often encompass diverse cranial pathologies, despite differing mechanisms of POH depending on the underlying condition. There is a lack of large-scale investigations focusing exclusively on POH following surgery for intracranial tumors. This study aimed to assess demographic variables—age, sex, and blood type—and pre-existing medical conditions as potential risk factors for POH in this specific context. **Methods:** A retrospective review was conducted on medical records of 1862 adult patients who underwent primary surgical resection of intracranial tumors. Univariate and multivariate analyses were applied to identify associations between POH and demographic or clinical characteristics. **Results**: POH, defined as postoperative hematoma necessitating surgical evacuation, was observed in 31 patients (1.7%). Univariate analysis revealed no statistically significant correlation between POH and demographic factors (age, sex) or pre-existing conditions such as hypertension, diabetes mellitus, cardiac disease, or liver dysfunction. **Conclusions:** The study found no evidence that demographic variables or pre-existing medical conditions independently contribute to the risk of POH following intracranial tumor resection in adults.

## 1. Introduction

POH is a rare complication following cranial neurosurgical procedures, often leading to severe neurological impairment or even death. In patients where cerebral hemorrhage causes increased intracranial pressure (ICP), surgical removal of the hematoma can be performed, but the outcomes may still be devastating. The reported mortality rate associated with increased ICP is approximately 30% [1,2]. In addition, POH has been reported as the most common cause of death following brain surgery [1,2].

POH can lead to increased intracranial pressure, edema, local or global cerebral ischemia, and life-threatening brain herniation. There are some studies describing the incidence and risk factors for the development of hemorrhage after craniotomy. Some reports suggest an association with age, pre-existing conditions, intraoperative and postoperative hypertension, and other comorbidities [3,4,5].

Major POHs requiring reoperation are rare, with an incidence rate between 1 and 3 percent [6,7,8]. However, smaller amounts of blood are frequently observed in connection with the surgical approach and resection cavity, but little is known about their potential clinical significance. Studies on larger postoperative hematomas have identified age [8,9] and tumor size [8] as possible risk factors.

Several factors may negatively influence the occurrence of POH, including disorders of various coagulation factors, perioperative and postoperative hypertensive episodes, type and location of the intracranial pathology, age, and similar variables [4,10,11].

The surgeon’s experience could have an influence on the risk of POH. Interestingly, some studies found a slightly higher incidence of POH when surgeons with many years of experience performed the surgery [12].

Peck et al. highlighted that the age of the patient should be considered in POH [13]. Hylek and Singer showed that age was the only significant independent risk factor for the development of subdural hemorrhage [14]. Previous studies found no significant association between baseline epidemiological factors and postoperative hemorrhage. In glioblastoma [15] and meningioma [16] surgery patients, variables such as age, sex, and body mass index (BMI) did not differ significantly between those with and without postoperative bleeding [15,16].

Basali et al. proposed that patients who developed POH were more likely to have hypertensive episodes in the intraoperative and/or early postoperative phase [4].

It is important to note that the factors associated with POH may vary depending on the intracranial disease. Therefore, this study was designed to investigate the preoperative risk factors for POH after intracranial surgery to help neurosurgeons identify patients at high risk for POH and weigh the risks of surgery against its putative benefits.

Our study aimed to clarify the extent to which intracranial hemorrhage after craniotomies was related to pre-existing medical conditions. In addition, demographic parameters such as patient age, sex, blood group, and Rhesus factor were taken into account. Bivariate and multivariate analysis methods were used to assess the influence of these parameters on the risk of developing POH.

## 2. Materials and Methods

The medical records and radiological images of patients who underwent craniocerebral surgery at our institution between 2008 and 2018 were analyzed retrospectively. During this period, more than 7100 patients underwent surgery for intracranial pathologies, of which 1862 procedures were performed for tumor resection. The retrospective review included various patient data such as age, sex, blood group, body mass index (BMI), perioperative ASA physical status classification, hypertension, diabetes, smoking history, cardiovascular disease, kidney disease, chronic inflammation, laboratory values, and the occurrence of POH.

Unless contraindicated, preoperative and postoperative imaging was performed using contrast-enhanced MRI. Steroids were administered preoperatively in cases of tumor-associated edema or significant mass effect. During surgery, intraoperative tools included ultrasound, electrophysiological monitoring, and, when necessary, frameless neuronavigation.

Exclusion criteria were age under 18 years and pregnancy. To statistically assess the potential influence of demographic characteristics and pre-existing conditions on POH, Fisher’s exact test and the Mann–Whitney U test were applied.

All postoperative imaging studies were carefully reviewed for signs of hemorrhage by two independent physicians. Significant postoperative hemorrhage was defined as hemorrhage that caused neurological symptoms such as increased intracranial pressure or mass effect requiring surgical intervention. Symptoms included focal neurological deficits, headache, nausea or vomiting, and cognitive changes.

Statistical analysis: In this study, categorical variables were summarized as absolute numbers and percentages. Continuous variables were described using medians and interquartile ranges (IQR), as none followed a normal distribution according to the Kolmogorov–Smirnov test. Associations between categorical variables and the occurrence of POH were evaluated using the chi-square test. When expected frequencies in contingency tables were below 5, Fisher’s exact test was applied instead. To compare continuous variables between patients with and without POH, the non-parametric Wilcoxon Mann–Whitney test was used. Variables that demonstrated statistical significance in univariate analyses were subsequently included in a multivariate logistic regression model to assess their independent association with POH. Variables with skewed distributions were log-transformed prior to inclusion in the regression model. All statistical analyses were carried out using SAS University Edition version 9.4 (SAS Institute Inc., Cary, NC, USA) and SPSS version 18.0 for Windows (SPSS Inc., Chicago, IL, USA). A two-tailed *p*-value of less than 0.05 was considered to indicate statistical significance.

## 3. Results

**Figure 1:**
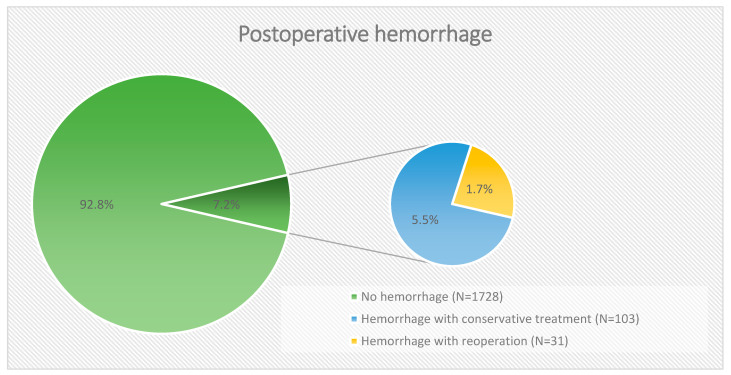
Classification of patients with postoperative hemorrhage into conservative and surgically treated patients.

In the period mentioned above, 1862 patients underwent intracranial tumor resection and 134 (7.2%) patients developed a hemorrhage. Of the operated patients, 31 (1.7%) developed POH, requiring a revision surgery with hematoma evacuation (Table 1). Figure 1 illustrates this subclassification of patients with postoperative hemorrhage as a circle diagram for improved clarity.

**Figure 2:**
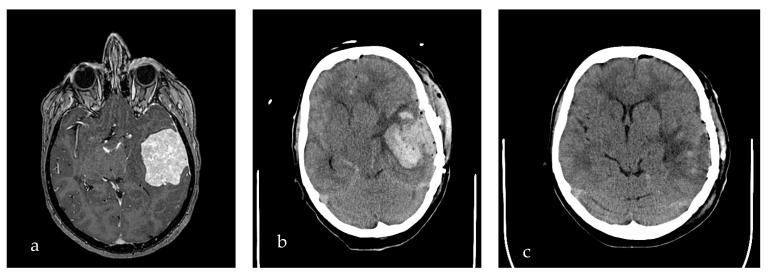
Sample postoperative hemorrhage in the resection cavity, necessitating revision surgery in the early postoperative period. (**a**) Contrast T1-WI preoperative image of left temporal meningioma. (**b**) CT-image depicting intraparenchymal hemorrhage in the resection cavity. (**c**) Follow-up CT-image two days after surgical hematoma evacuation.

Figure 2 shows a typical example of POH in the resection cavity, requiring reoperation and hematoma evacuation because of its space-occupying character and midline shift on postoperative CT-images.

### 3.1. Predictors of Hemorrhagic Complications

**Figure 3:**
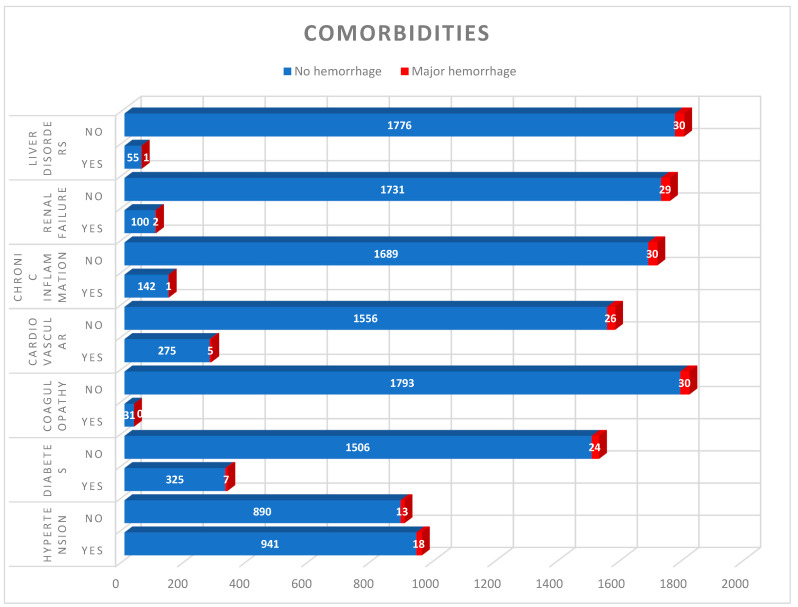
Comorbidities and their association with clinically significant POH. None of the pre-existing conditions observed was found to have a statistically significant influence on the occurrence of POH.

We investigated possible predictors of hemorrhagic complications in the entire study cohort. First, we examined the effects of demographic characteristics and major comorbidities using univariate analysis (Table 2 and Figure 3).

#### 3.1.1. Demographic Characteristics

Demographic characteristics and additional patient data such as sex (*p* = 0.857), blood type (*p* = 0.520), Rh-factor (*p* = 1.000), smoking status (*p* = 0.419), patient age at the time of surgery (*p* = 0.755), BMI (*p* = 0.482), and ASA score (*p* = 0.214) showed no significant correlation.

#### 3.1.2. Comorbidities and Tumor Characteristics

Arterial hypertension (*p* = 0.476), diabetes mellitus (*p* = 0.478), heart disease (*p* = 0.801), dyslipoprotein (*p* ≤ 0.01), renal insufficiency (*p* = 0.686), coagulopathy (*p* = 1.000), chronic inflammation (*p* = 0.509), and liver disease (*p* = 0.615) showed no influence on the postoperative bleeding risk (Figure 3). Tumor characteristics included re-operation (*p* = 0.637), tumor type (*p* = 0.972) were also evaluated. Apart from the parameters listed, there was no significant correlation (Table 3).

#### 3.1.3. Laboratory Parameters

Laboratory parameters such as C-reactive protein (CRP) (*p* = 0.106), complete blood counts such as white blood cell count (*p* = 0.071) and platelet count (*p* = 0.404), and coagulation parameters such as international normalized ratio (INR) (*p* = 0.202) and partial thromboplastin time (PTT) (*p* = 0.816) also showed no significant association with POH (see Table 4).

## 4. Discussion

The incidence of POH following cranial neurosurgery shows considerable variability in the literature, largely due to differences in definitions, patient populations, and surgical contexts. Reported rates typically range from below 1% to approximately 4%, with higher figures observed in specific cohorts or high-risk groups [3,12,15,16,17,18,19,20]. In our patient cohort, the incidence of clinically relevant POH—defined as hemorrhage requiring surgical evacuation—was 1.7% (*n* = 31), which is consistent with the lower to mid-range values reported across neurosurgical series.

Differences in reported POH rates also reflect varying surgical indications and underlying pathologies. For instance, Desai et al. noted that the majority of postoperative hemorrhages were subdural or intraparenchymal in nature [21], whereas other authors have identified epidural or resection cavity-associated intraparenchymal hematomas as more common [4,12,20,22,23]. These anatomical variations likely depend on the type of surgery performed and the characteristics of the preoperative lesion, underscoring the importance of contextualizing POH risk within the broader neurosurgical spectrum [21].

Although POH has been documented in the literature, relatively few studies have explored its risk factors in depth. While some studies identified selected demographic, clinical, and surgical variables associated with POH, a comprehensive and current analysis of these potential contributors remains lacking.

Demographic variables such as age and sex have been extensively studied as potential risk factors for POH, but the evidence is inconsistent. Some studies suggest that advancing age may increase bleeding risk due to vascular fragility or comorbid conditions. For example, some studies including the study by Wang et al. and Palmer et al. identified older age as an independent risk factor for POH following cranial tumor resection [2,8]. However, other studies, including those by Greuter et al. [24] and Wilhelmy et al. [15], did not observe any statistically significant associations between patient age or sex and the occurrence of POH [17,25]. Our own findings align with the latter group: neither age (*p* = 0.755) nor sex (*p* = 0.857) was significantly associated with POH in our cohort. These results are consistent with much of the current literature and reinforce the view that demographic characteristics alone are not reliable predictors of postoperative bleeding in neurosurgical patients. While male patients often predominate in neurosurgical studies [20,26], some cohorts report a higher proportion of female participants [27]; however, no consistent evidence supports a clinically meaningful relationship between sex and POH risk. Another possible explanation for the current results could be the underlying mechanism of blood clotting, which is generally considered to be independent of sex or age.

Diabetes mellitus has been explored as a potential risk factor for postoperative bleeding due to its effects on vascular integrity and wound healing. Abouzari et al. hypothesized that hyperosmolar states in diabetic patients might promote hematoma expansion through fluid shifts into the developing clot [28]. In contrast, Yamamoto et al. proposed that elevated blood viscosity secondary to enhanced coagulation activity in diabetes could reduce bleeding risk [29]. These opposing theories illustrate the complexity of pathophysiological mechanisms in diabetic patients. Despite these theoretical considerations, empirical data remain inconclusive. Wang et al. [8] found no significant association between diabetes or other metabolic conditions such as hypercholesterolemia or hypertension and the risk of POH after intracranial tumor resection. Other large-scale studies similarly reported no differences in postoperative bleeding rates between diabetic and non-diabetic patients [6]. Our analysis corroborates these findings; as we did not observe any statistically significant association between diabetes and POH (*p* = 0.478), further questioning the clinical relevance of diabetes in this context. These findings could also be explained with the medical treatment of these

Atherosclerotic disease, including cardiac, cerebrovascular, and peripheral vascular conditions, may theoretically increase the risk of intracranial hemorrhage by compromising vascular autoregulation. Gudeman et al. postulated that elevated systemic pressure could be transmitted to the cerebral microvasculature, weakening vascular walls and promoting hemorrhage formation [30]. Nonetheless, clinical evidence supporting this theory remains inconsistent. Hypertension is a well-established risk factor for spontaneous intracerebral hemorrhage and has been implicated in perioperative bleeding due to its effects on cerebral autoregulation and hemostasis [4]. Nevertheless, its role in POH remains ambiguous. Acute elevations in blood pressure particularly when the blood–brain barrier has been disrupted by surgery may lead to extravasation of blood into the resection cavity [31]. Additionally, high systemic pressure may impair clot formation and promote hematoma development [5,31]. While some studies have demonstrated an association between perioperative hypertension and hematomas requiring surgical evacuation [3,8], others have not substantiated this link [4]. Some studies, such as those by Basali and Kwinta et al., have identified hypertension, ischemic heart disease, and atrial fibrillation as contributors to early postoperative reoperation, often linked to anticoagulant use [4,32]. However, other large-scale investigations, such as the study by Wang et al., failed to confirm a direct association between these conditions and POH risk [8]. In our study, we observed no significant relationship between hypertension (*p* = 0.476), cardiovascular disease (*p* = 0.801), or any other pre-existing cardiovascular comorbidity and the incidence of POH. These findings support the view that while cardiovascular conditions may influence overall perioperative risk, their specific role in POH remains uncertain. Due to high awareness among medical staff and strict treatment protocols, these comorbidities played an irrelevant role in postoperative hemorrhagic complications.

Effective surgical hemostasis requires normal platelet function, intact coagulation pathways, and minimal fibrinolytic activity. Standard and extended laboratory parameters can predict the risk of POH [2]. Low platelet counts particularly below 100,000/μL have been associated with increased bleeding risk [2], and Chan et al. demonstrated that even moderate postoperative declines in platelet levels can predispose patients to hematoma formation [33]. Wagner et al. emphasized the importance of advanced preoperative screening, including factor XIII (FXIII), von Willebrand factor (vWF), and platelet function analysis (PFA), along with standard coagulation markers such as partial thromboplastin time (PTT) [17]. Their study demonstrated that correcting deficiencies in these factors reduced the rate of postoperative hematomas requiring surgical evacuation. In contrast, Greuter et al. analyzed more conventional laboratory parameters including preoperative hemoglobin, platelet count, and international normalized ratio (INR) and found no significant differences between patients with and without POH [24]. Our findings are in agreement with the latter study, since routine laboratory values, including coagulation factors, did not show any statistically significant association with POH in our cohort. However, it is important to note that our study did not incorporate the extended coagulation diagnostics employed in Wagner’s protocol, which may explain the discrepancy in predictive value. Another relevant point is the missing follow-up laboratory data, which could have strengthened the current results.

## 5. Conclusions

This study is limited by its retrospective, single-center design, which may introduce selection bias and limit generalizability. Future prospective multicenter studies with larger and more diverse patient populations are warranted to address these limitations and improve statistical power, particularly for subgroup analyses. Our statistical analyses did not identify any significant associations between POH and demographic variables such as age or sex, nor with pre-existing medical conditions including diabetes mellitus, hypertension, or cardiovascular disease. Similarly, routine laboratory parameters failed to demonstrate predictive value for POH in this cohort. The multifactorial etiology of POH, coupled with the lack of consistent associations across commonly assessed variables, underscores the need for further research. Future studies should incorporate standardized definitions of POH, advanced coagulation profiling, and comprehensive preoperative risk models to enhance patient stratification and optimize perioperative management strategies.

## Figures and Tables

**Table 1:** Incidence of postoperative hemorrhage following cranial neurosurgery. A total of 103 patients (7.2%) experienced postoperative rebleeding, of whom 31 patients (1.7%) required reoperation due to either mass effect caused by the hemorrhage or the development of new neurological deficits.

Hemorrhage	*n*	%
No postoperative bleeding	1728	92.8
Bleeding without reoperation	103	5.5
Bleeding with reoperation	31	1.7
Total	1862	100

**Table 2:** Summary of key demographic variables and their association with POH. None of the demographic factors demonstrated a statistically significant impact on the incidence of POH.

Demographic Data		No Hemorrhage *n* (%)	Hemorrhage with Reoperation *n* (%)	*p*-Value
Sex	female male	935 (51.06) 896 (48.94)	15 (48.39) 16 (51.61)	0.857
Age [years]—mean ± SD		59.8 ± 15.6	60.6 ± 14.9	0.755
BMI—mean ± SD		27.5 ± 5.5	28.2 ± 4.8	0.482
Blood group	A B AB 0	753 (43.30) 224 (12.88) 115 (6.61) 647 (37.21)	11 (35.48) 3 (9.68) 1 (3.23) 16 (51.61)	0.520
Rh-factor	negative positive	310 (17.85) 1427 (82.15)	5 (16.13) 26 (83.87)	1.000
ASA-Score	I II III IV	100 (5.49) 1024 (56.26) 649 (35.66) 47 (2.58)	0 (0.00) 15 (50.00) 13 (43.33) 2 (6.67)	0.214
Smoker	yes no	473 (26.59) 1306 (73.41)	6 (19.35) 25 (80.65)	0.419

**Table 3:** Depicting general tumor characteristics in correlation to hemorrhage during the postoperative period.

Parameters			Hemorrhage		
			No Hemorrhage *n* (%)	Hemorrhage with Operation *n* (%)	*p*-Value
**Tumor** **characteristics**	Recurrence	Yes No	315 (17.37) 1498 (82.63)	4 (12.90) 27 (87.10)	0.637
	Diagnostic group	Glioma Meningioma Metastases other	642 (35.06) 414 (22.61) 359 (19.61) 416 (22.72)	10 (32.26) 8 (25.81) 6 (19.35) 7 (22.58)	0.972

**Table 4:** Laboratory parameters and their association with POH. No laboratory values demonstrated a statistically significant correlation with the occurrence of POH.

Laboratory Parameters	No Hemorrhage	Hemorrhage with Reoperation	*p*-Value
	Mean [Scatter Range]	Mean [Scatter Range]	
INR	0.99 [0.99; 1.00]	1.02 [1.02; 1.03]	0.202
PTT [s]—mean ± SD	27.4 ± 3.6	27.3 ± 3.6	0.816
Platelets [10^9^/L]—mean ± SD	269.9 ± 90.3	250.7 ± 125.8	0.404
C-reactive protein [mg/L]	5.3 [0.4; 10.2]	3.1 [0.0; 6.2]	0.106
Leukocytes [10^9^/L]	10.8 [10.2; 11.5]	9.2 [8.8; 9.5]	0.071

## Data Availability

The datasets obtained and analyzed during the current study are available from the corresponding author on reasonable request. The data are not publicly available due to ethical restrictions.

## Abbreviations

The following abbreviations are used in this manuscript:

POH	Postoperative hemorrhage
BMI	Body-mass-index
ASA	American Society of Anesthesiologists
ICP	Intracranial pressure
MRI	Magnetic resonance tomography
CT	Computer tomography
CRP	C-reactive protein
INR	International normalized ratio
PTT	Partial thromboplastin time
DM	Diabetes mellitus
